# Supplementary figures and images for: Knockout of ribosomal protein RpmJ leads to zinc resistance in Escherichia coli
Source: PLoS One. 2023 Mar 24;18(3):e0277162. doi: 10.1371/journal.pone.0277162 (PMC10038286; doi:10.1371/journal.pone.0277162)

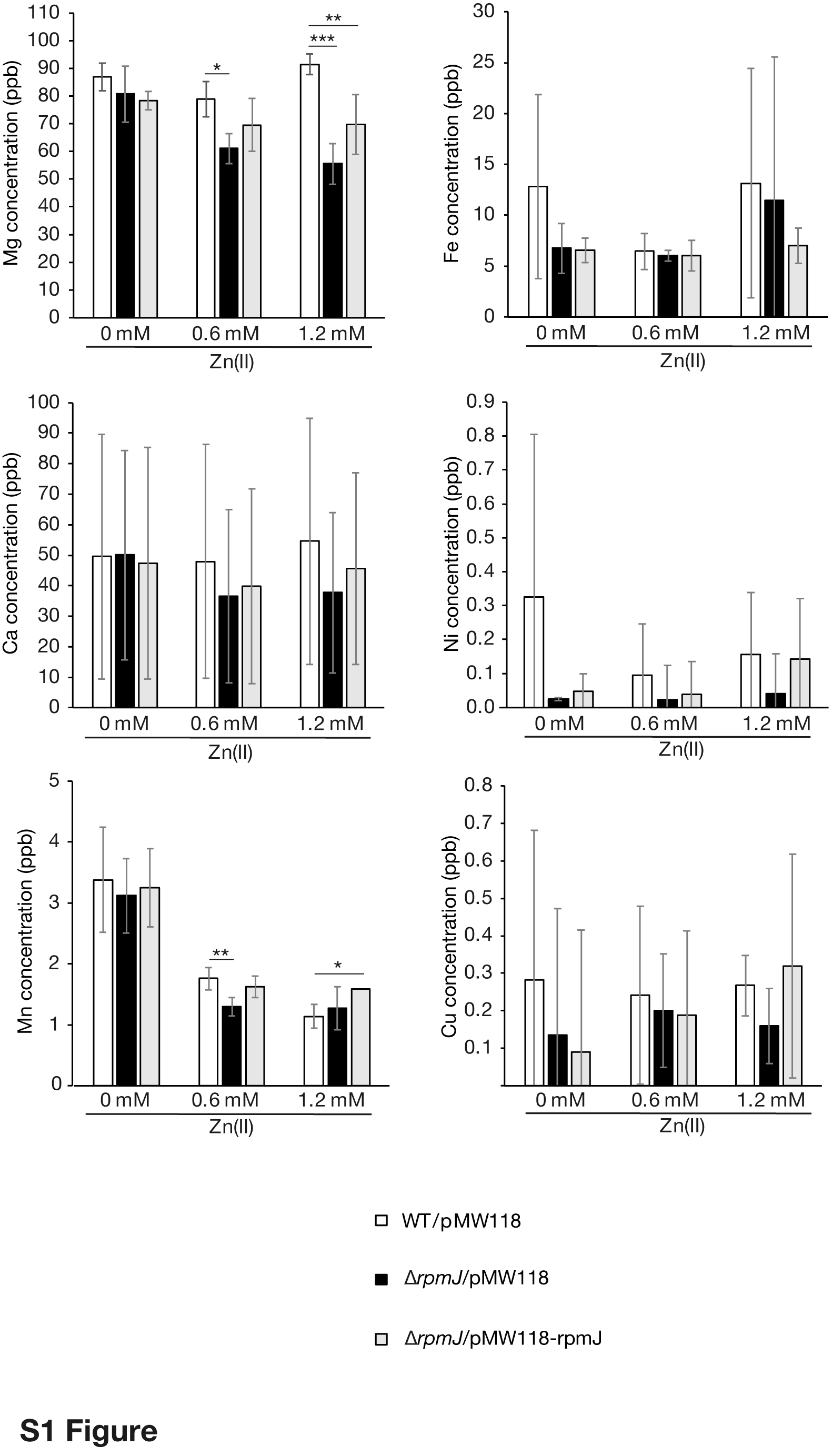

Supplement: S1 Fig — Wild-type E. coli strain transformed with an empty vector (WT/pMW118), the rpmJ mutant transformed with an empty vector (ΔrpmJ/pMW118), the rpmJ mutant transformed with a plasmid carrying an intact rpmJ gene (ΔrpmJ/pMW118-rpmJ) were cultured under conditions of 0 mM Zn(II), 0.6 mM Zn(II), or 1.2 mM Zn(II), in the presence of 1mM IPTG. The metal concentrations were measured by ICP-MS. Data shown are means ± standard deviation from 4 independent experiments (*, p value <0.05, **, p value <0.01, ***, p value <0.001). (TIF) [file pone.0277162.s002.tif]
